# Supplementary figures and images for: Mining of candidate genes involved in the biosynthesis of dextrorotatory borneol in Cinnamomum burmannii by transcriptomic analysis on three chemotypes
Source: PeerJ. 2020 Jun 10;8:e9311. doi: 10.7717/peerj.9311 (PMC7293187; doi:10.7717/peerj.9311)

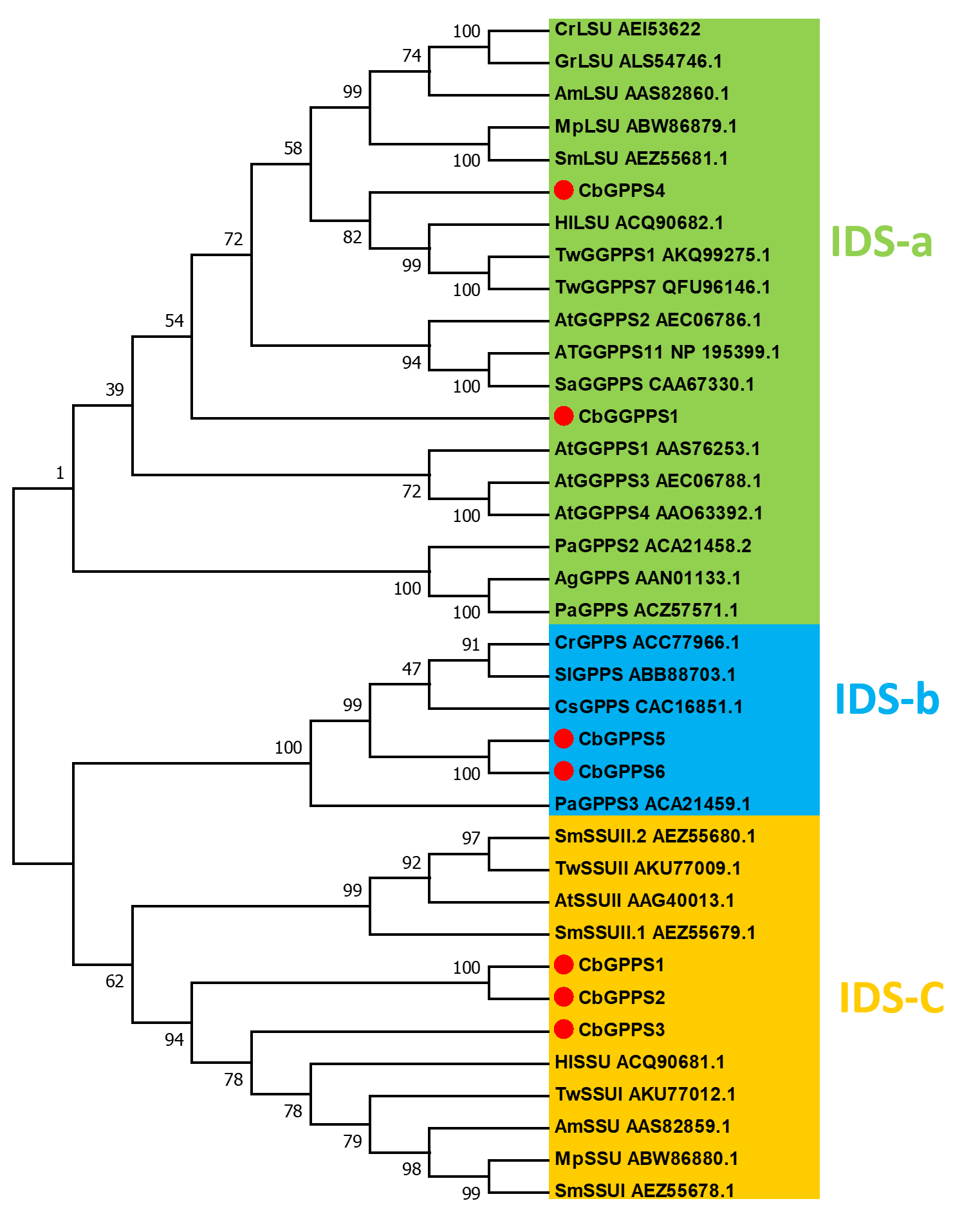

Supplement: Figure S1 [file peerj-08-9311-s020.png]

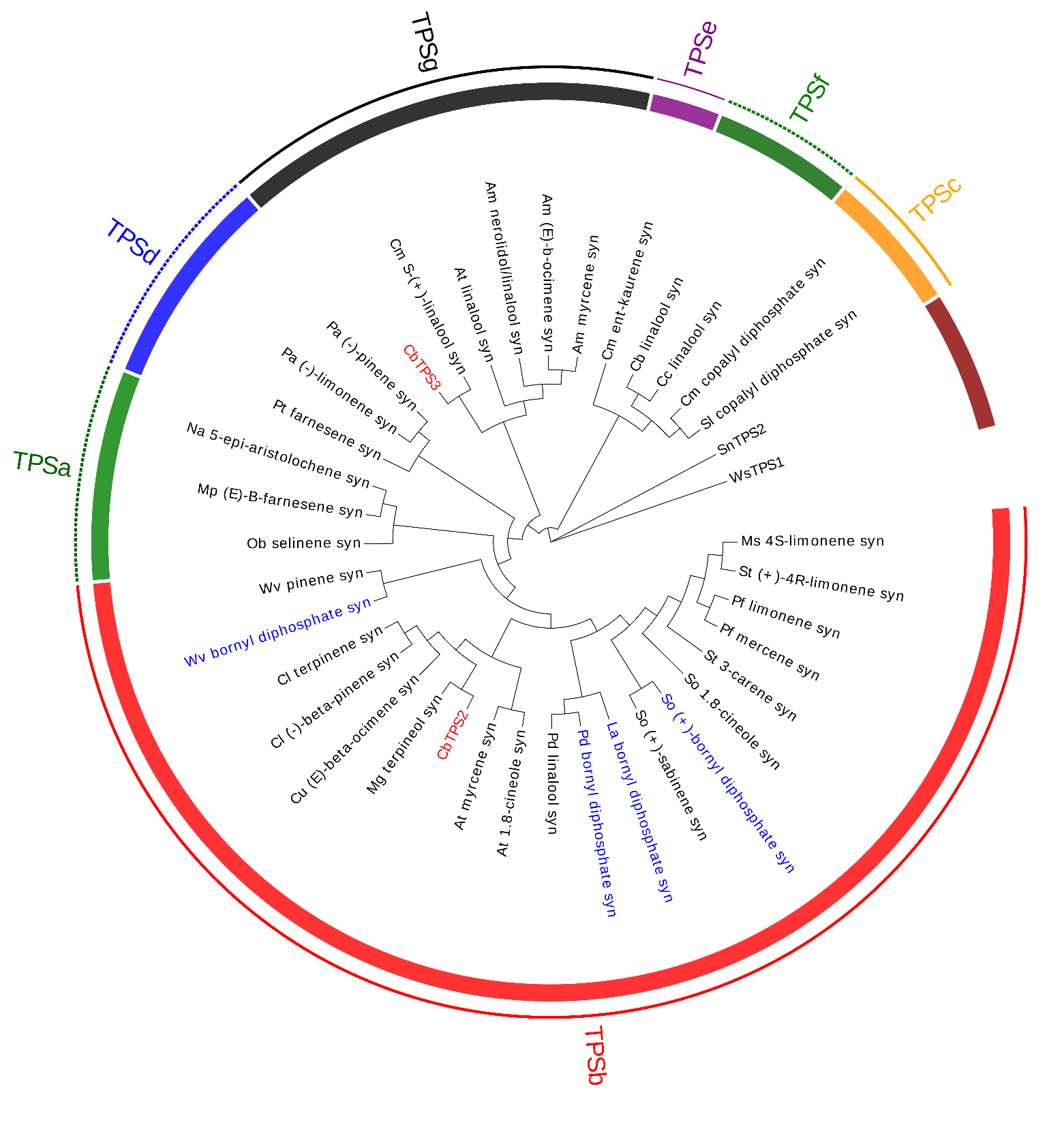

Supplement: Figure S2 — CbTPS2 and CbTPS3 were marked in red. The four functionally characterized BPPS were shown in blue font. Abbreviation and NCBI accession numbers are provided in Supplemental Data 18. [file peerj-08-9311-s021.png]

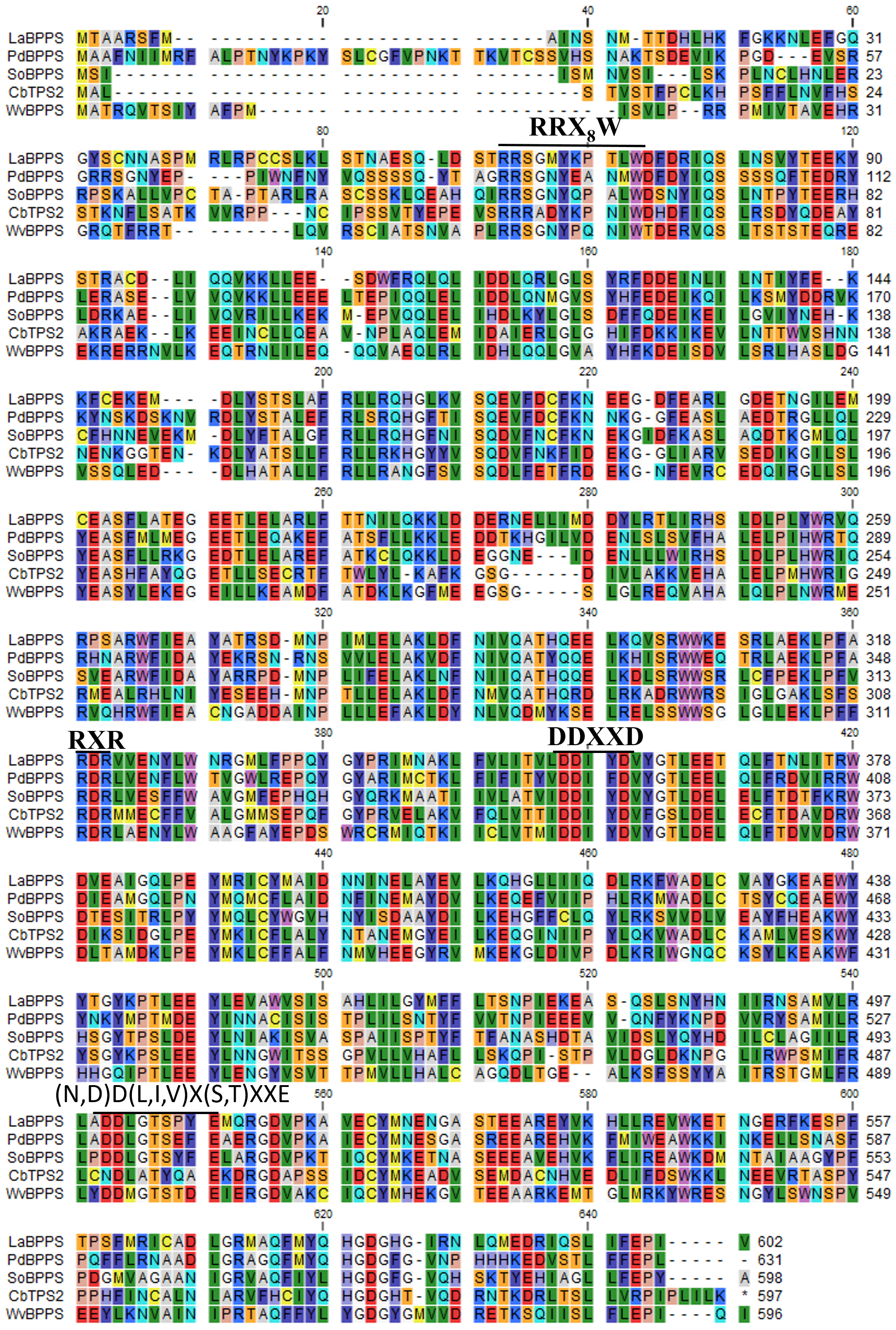

Supplement: Figure S3 — LaBPPS: La bornyl diphosphate synthase. SoBPPS: So (+)-bornyl diphosphate synthase; PdBPPS: Pd bornyl diphosphate synthase; WvBPPS: Wv bornyl diphosphate synthase. [file peerj-08-9311-s022.png]

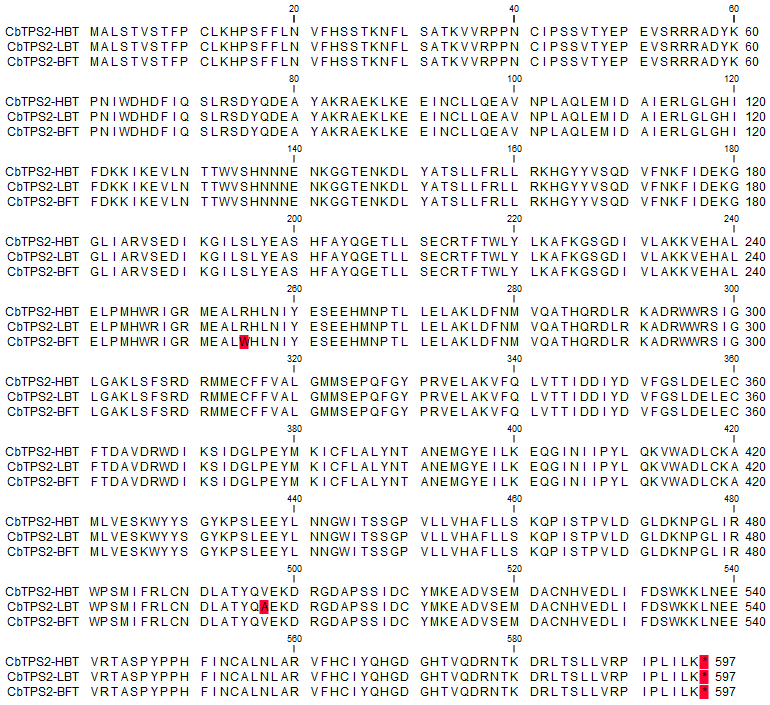

Supplement: Figure S4 [file peerj-08-9311-s023.png]

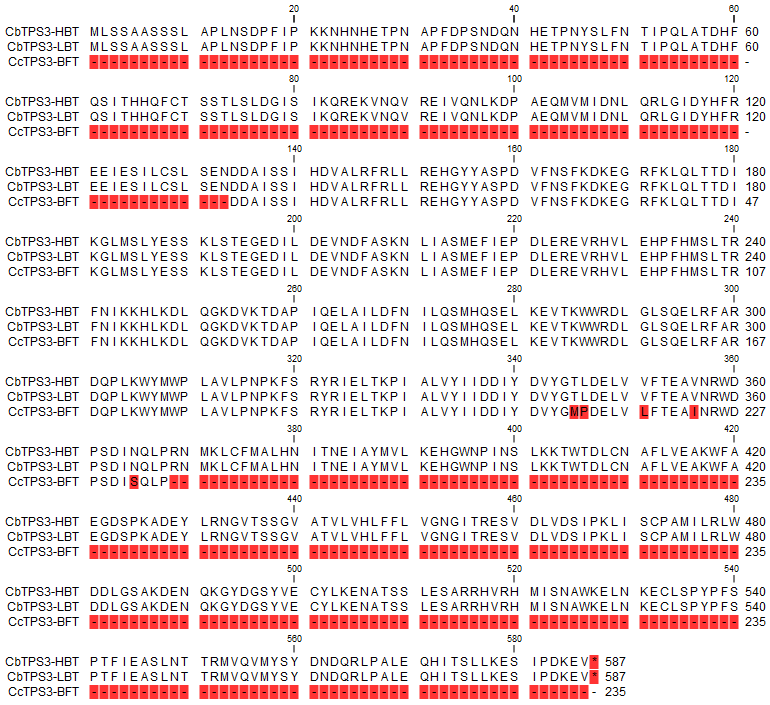

Supplement: Figure S5 [file peerj-08-9311-s024.png]
